# Personalised neoantigen vaccines for precision oncology in Southeast Asia: clinical opportunities, infrastructure challenges, and policy perspectives

**DOI:** 10.3389/fimmu.2026.1934309

**Published:** 2026-09-11

**Authors:** Yingying Gao, Yuan Wang, Junwei Li

**Affiliations:** Department of Infectious Diseases, The Second Hospital of Nanjing, Affiliated to Nanjing University of Chinese Medicine, Nanjing, China

**Keywords:** cancer health equity, genomic infrastructure, personalised neoantigen vaccines, precision oncology, Southeast Asia (ASEAN), translational policy

## Abstract

Personalised neoantigen vaccines are patient-specific immunotherapies designed to target tumour-restricted antigens arising from genomic and transcriptomic alterations, including single-nucleotide variants, insertions and deletions, frameshifts, gene fusions and aberrant splicing. Early clinical studies have established manufacturing feasibility and immunogenicity, while randomised phase 2b evidence in resected melanoma has provided a signal of clinical activity that requires confirmation in larger trials. Clinical development nevertheless remains concentrated in high-income academic centres, raising questions about equitable implementation in Southeast Asia. This narrative review evaluates the published clinical evidence and the documented health-system capacity of the 11 ASEAN member states. Regional capacity is heterogeneous: national or institutional genomic programmes and tertiary molecular services are documented in several countries, whereas routine nationwide availability, public reimbursement and equitable geographic access remain incompletely characterised. Potential implementation barriers include access to clinical-grade tumour–normal sequencing, bioinformatics and molecular pathology expertise, patient-specific GMP manufacturing, chain-of-identity control, product release testing, temperature-controlled distribution, financing and country-specific regulatory requirements. Current immunopeptidomic training datasets provide uneven HLA coverage, and published neoantigen-prediction tools have not undergone prespecified, prospective, allele-stratified validation across representative Southeast Asian HLA class I and class II repertoires. Their regional transportability should therefore be considered uncertain rather than assumed to be either equivalent or inferior. A staged hub-and-spoke model linking clinical centres, national genomic nodes and a limited number of regional GMP facilities could support prospective implementation studies. Whether personalised neoantigen vaccines reduce or widen existing cancer inequities will depend on evidence-based patient selection, health-system investment, sustainable financing and nationally appropriate regulatory oversight.

## Introduction

Cancer has become a defining public health crisis of the 21st century, with a disproportionate burden shifting toward low- and middle-income countries (LMICs). Southeast Asia, home to over 680 million people, exemplifies this transition: Dee et al., 2025 (1) documented more than 1.1 million new cancer diagnoses and approximately 700 000 cancer-related deaths in the region in 2022. The epidemiological transition is further characterised by profound disparities in incidence and survival linked to socioeconomic status, geography, and ethnicity (1), while oncology infrastructure remains fragmented and skewed toward urban centres, leaving large populations without timely access to even basic diagnostic and therapeutic services (2).

Precision oncology uses molecular, genomic and other biomarkers to guide prevention, diagnosis, prognosis and treatment for an individual patient; it is broader than cancer immunotherapy. In this Review, a personalised neoantigen vaccine is a patient-specific product manufactured from tumour-derived antigen candidates identified in that individual. Individualised neoantigen therapy is the broader category of patient-specific neoantigen-directed interventions, including vaccines and cellular approaches, whereas a shared neoantigen vaccine targets a recurrent antigenic alteration present in more than one patient within a compatible HLA context. Personalised neoantigen vaccines aim to prime or expand T cells against tumour-selective epitopes while limiting recognition of normal tissues (3, 4). Their development integrates tumour–normal sequencing, RNA-expression analysis, HLA typing, computational prioritisation and adaptable peptide or nucleic-acid manufacturing. Computational models narrow the candidate space, but prediction is an enrichment step rather than a substitute for immunopeptidomic or T-cell validation (5, 6).

Despite this revolution, Southeast Asia confronts a unique intersection of obstacles that must be addressed before personalised neoantigen vaccines can become a clinical reality. Major barriers—limited genomic infrastructure, workforce shortages, regulatory fragmentation, and financial inequities—continue to restrict access to precision oncology across much of the region (7–10). These interconnected deficits represent a systemic bottleneck that cannot be overcome by technology alone.

This narrative review synthesises recent literature on personalised neoantigen vaccines, precision oncology implementation, and healthcare systems challenges in Southeast Asia, drawing on evidence from PubMed, Scopus, and Web of Science.

Despite the burgeoning global literature, no synthesis has yet examined the confluence of clinical, infrastructural, and policy factors that will determine the feasibility of neoantigen vaccines in Southeast Asia. Mateo et al., 2022 (11) have argued that delivering precision oncology equitably requires not only scientific breakthroughs but also health-system strengthening, sustainable financing, and robust governance. By synthesising clinical evidence, technological foundations, and implementation barriers, this review aims to inform clinicians, health ministries, and international partners, and to catalyse a regional dialogue on the responsible introduction of next-generation cancer immunotherapies.

## Precision oncology landscape in Southeast Asia

### Cancer epidemiology and burden

The epidemiological transition sweeping across LMICs has created a “double burden” of infection-related and non-communicable cancers in Southeast Asia. Lung cancer remains the leading cause of cancer death, with unique epidemiological features in the region (12). Hepatocellular and cervical cancers, linked to endemic hepatitis B and human papillomavirus, add substantially to the burden (13). Access to cancer screening, molecular diagnostics, and advanced therapeutics remains highly unequal; even in relatively well-resourced settings, screening services are not equitably distributed (14).

### Virus-associated cancers and antigen selection

The regional burden of HBV-, HCV- and HPV-associated cancers changes the antigen landscape but should not be conflated with somatic neoantigens. HPV E6 and E7 are non-self, tumour-maintained viral antigens shared across many patients rather than patient-specific mutation products; the single-arm phase II study of the HPV16 E6/E7 synthetic long-peptide vaccine ISA101 plus nivolumab in 24 patients with incurable HPV16-positive cancer reported an objective response rate of 33%, providing a clinical precedent for combining a shared viral-antigen vaccine with checkpoint blockade (15). In HBV-associated hepatocellular carcinoma, integrated HBV DNA can encode expressed epitopes recognised by T cells, and tumour-resident HBV-specific T-cell states have been associated with relapse-free survival (16, 17). These targets may complement somatic neoantigens, but candidate selection must confirm tumour expression and consider off-tumour recognition of infected nonmalignant hepatocytes. HCV is a positive-sense RNA virus that does not integrate into the host genome; therefore, HCV-associated hepatocarcinogenesis does not provide the same stable, tumour-integrated viral target class. In HCV-associated disease, chronic inflammation, cirrhosis and immune dysfunction may be more relevant to vaccine response than persistent tumour-specific HCV antigen expression. Regional trials should prospectively stratify viral aetiology and evaluate viral-antigen and somatic-neoantigen responses separately.

### Current status of precision oncology

Precision oncology has made inroads into Southeast Asia in a highly fragmented and inequitable fashion. Next-generation sequencing (NGS) capacity is expanding in selected academic centres but remains limited in routine public-sector oncology care. In Thailand, NGS utilisation is confined largely to research settings and a few tertiary referral centres, with minimal penetration into provincial hospitals (18). Biomarker-guided therapies and molecular testing remain inconsistently available across public hospitals (8). Singapore represents a regional leader in genomic medicine, although disparities in biomarker access persist across healthcare settings (19). In Cambodia, even basic oncology infrastructure is nascent, as illustrated by the initial cohort of cervical cancer patients treated at the National Cancer Centre (20). Overall, precision oncology infrastructure across Southeast Asia remains fragmented, urban-centred, and inaccessible to many patients (**Table 1**).

**Table 1:** Country-level evidence on genomics and precision-oncology capacity in Southeast Asia.

Country	Documented activity or capacity	Evidence-based interpretation	Evidence limitations and citations
Singapore	The PRECISE-SG100K programme has generated whole-genome sequencing data from approximately 100,000 Singaporean residents, while substantial tertiary clinical and cancer-research capacity is documented.	Singapore has the most extensively documented national precision-medicine ecosystem in Southeast Asia. However, population-genomics capacity does not demonstrate routine, reimbursed comprehensive genomic profiling for all patients with cancer.	Routine oncology use and reimbursement require service- and payer-specific evidence (19, 21).
Thailand	Genomics Thailand supports national whole-genome sequencing infrastructure and cancer-related genomic research, while NGS capability is established within academic and tertiary centres.	Thailand has substantial research and tertiary-care capability, although routine availability, reimbursement and implementation outside major centres remain incompletely documented.	Evidence primarily reflects national initiatives and tertiary or research settings rather than uniform implementation across the public health system (18).
Malaysia	National precision-medicine and genome-sequencing initiatives, together with academic genomic research and diagnostic activity, are documented.	Malaysia has growing national genomic capability, but the available evidence does not establish universal access to clinical oncology NGS or nationwide reimbursement.	Published evidence describes emerging national capacity rather than routine, equitable implementation across all oncology services (22)
Indonesia	The Biomedical and Genome Science Initiative includes whole-genome sequencing for cancer and other priority diseases across designated national referral hospitals. National health insurance has also expanded access to basic cancer services.	National investment and referral-centre genomic capacity are established; however, routine publicly financed oncology NGS and equitable geographic access have not been demonstrated nationally.	Evidence is strongest for national programmes and selected referral hospitals and should not be generalised to all provinces or public facilities (7, 10, 23)
Vietnam	Primary research has generated a somatic-variant dataset from Vietnamese women with breast cancer, demonstrating local tumour-sequencing and molecular-analysis capability.	Tertiary and research-level tumour-genomics capacity is documented, but nationwide routine availability and reimbursement of oncology NGS have not been established.	The available evidence reflects specific research cohorts and cannot be generalised to routine national oncology practice (24)
Philippines	Cancer specialists, radiotherapy facilities and advanced cancer services are concentrated in Metro Manila and other major urban centres, with marked geographic barriers affecting access.	Specialist, academic and private-sector capacity exists, but the available studies do not demonstrate nationwide routine access to publicly financed oncology NGS.	The available studies primarily evaluate workforce and geographic access rather than the national availability or reimbursement of genomic profiling (25, 26)
Cambodia	The National Cancer Centre and expanding tertiary oncology services demonstrate continuing development of national cancer-care infrastructure.	Cancer-service development is documented, but the available evidence is insufficient to classify the nationwide availability of oncology NGS, molecular pathology or biobanking.	Evidence relates primarily to tertiary cancer-service development and broader ASEAN diagnostic disparities (7, 20).
Lao PDR	Regional evidence identifies substantial constraints in access to cancer diagnostics, while country-specific evidence on oncology-genomics infrastructure remains sparse.	The current evidence base does not support either nationwide availability or complete absence of NGS, molecular pathology or biobanking.	Country-specific oncology-genomics capacity remains an evidence gap in the reviewed literature (7).
Myanmar	Regional evidence identifies constraints in access to cancer diagnostics, while current country-specific mapping of oncology-genomics services is limited.	Current national capacity cannot be classified reliably, particularly because health-system disruption may affect the availability and documentation of services.	Country-specific oncology-genomics infrastructure remains insufficiently characterised in the reviewed literature (7).
Brunei Darussalam	The Precision Genomic Medicine Research Group and Biomedical Genomics Core Laboratory at Universiti Brunei Darussalam provide NGS research capacity and collaborate with national cancer services.	Institutional genomic-research capability is documented, but routine nationwide clinical oncology NGS and reimbursement remain uncharacterised.	Available evidence describes institutional research capacity and planned clinical translation rather than established nationwide implementation (7, 27)
Timor-Leste	Timor-Leste became ASEAN’s eleventh member on 26 October 2025. Sufficient country-specific evidence on oncology-genomics infrastructure was not identified in the reviewed literature.	National oncology-genomics capacity cannot currently be classified; the absence of published evidence should not be interpreted as proof that genomic or molecular diagnostic services are completely unavailable.	ASEAN membership is officially documented, but country-specific oncology-genomics evidence remains insufficient (28).

ASEAN, Association of Southeast Asian Nations; NGS, next-generation sequencing; PRECISE-SG100K, Precision Health Research Singapore 100,000 Genomes Project; WGS, whole-genome sequencing.

The table distinguishes documented research or tertiary-centre capacity from routine nationwide clinical availability and reimbursement. An evidence gap indicates that sufficiently specific published or official information was not identified; it does not establish the complete absence of a service.

### Healthcare disparities and access inequities

The inequitable distribution of oncology resources is the single most formidable barrier. A multi-country assessment documented extreme heterogeneity: while some urban centres possess PET-CT scanners and NGS platforms, many rural provinces lack even basic immunohistochemistry (7). In the Philippines, oncologists, pathologists, and advanced imaging facilities are overwhelmingly clustered in Metro Manila, leaving rural and island communities effectively excluded (25); similar geographic barriers affect radiotherapy access (26). Financial toxicity compounds these physical barriers. Despite national health insurance expansion in Indonesia, high out-of-pocket payments, restricted formularies, and inconsistent reimbursement for molecular tests mean that even insured patients frequently cannot afford comprehensive genomic profiling (10). These entrenched disparities mean that any strategy to introduce personalised neoantigen vaccines must be accompanied by deliberate health-system strengthening, innovative financing, and decentralised delivery models; otherwise, such technologies risk widening the chasm between the privileged few and the underserved many.

### Genomic diversity and population-specific considerations

HLA allele frequencies differ substantially across Southeast Asian populations, but ancestry itself is not an input to peptide–HLA predictors; performance depends on the representation of the relevant allele–peptide combinations, assay quality and the model’s ability to generalise. Training immunopeptidomic datasets are uneven across HLA alleles and populations. An independent evaluation of NetMHCpan-4.1 reported underrepresentation of alleles associated with Asian and Pacific Islander populations in training data but found only a small median decrement for alleles absent from training, illustrating why training imbalance should not automatically be described as failure in all Asian populations (29). The unresolved regional question is allele-stratified external validation across common Southeast Asian HLA-A, HLA-B, HLA-C, HLA-DR, HLA-DQ and HLA-DP repertoires using monoallelic or deconvoluted immunopeptidomics and orthogonal T-cell assays.

## Biological and technological foundations of personalised neoantigen vaccines

### Tumour neoantigens and cancer immunogenicity

Tumour-specific antigens can arise at several molecular levels. In addition to nonsynonymous single-nucleotide variants, small insertions and deletions—especially frameshifts—can create novel open reading frames; gene fusions can generate junctional peptides; and aberrant splicing can produce tumour-restricted neojunctions (30–32). RNA editing, noncanonical translation and selected noncoding events may provide additional candidate sources, but their tumour specificity and peptide presentation require rigorous validation. A genomic alteration becomes a vaccine-relevant neoepitope only if it is present in a sufficient fraction of tumour cells, transcribed and translated, processed, presented by the patient’s HLA molecules, and recognised by a functional T-cell repertoire.

Tumour mutational burden (TMB) counts mutations rather than antigen presentation or T-cell recognition. It can associate with checkpoint-inhibitor benefit in selected tumour types, but its predictive value is context dependent and it is not a direct measure of vaccine-relevant immunogenicity (33). Candidate quality also depends on clonality, mutant-allele expression, peptide processing and presentation, HLA restriction, similarity to self, and the available T-cell repertoire. TMB should therefore be described as one input to candidate discovery, not as a surrogate endpoint for neoantigen-vaccine activity.

### Workflow of personalised neoantigen vaccine development

**Figure 1:**
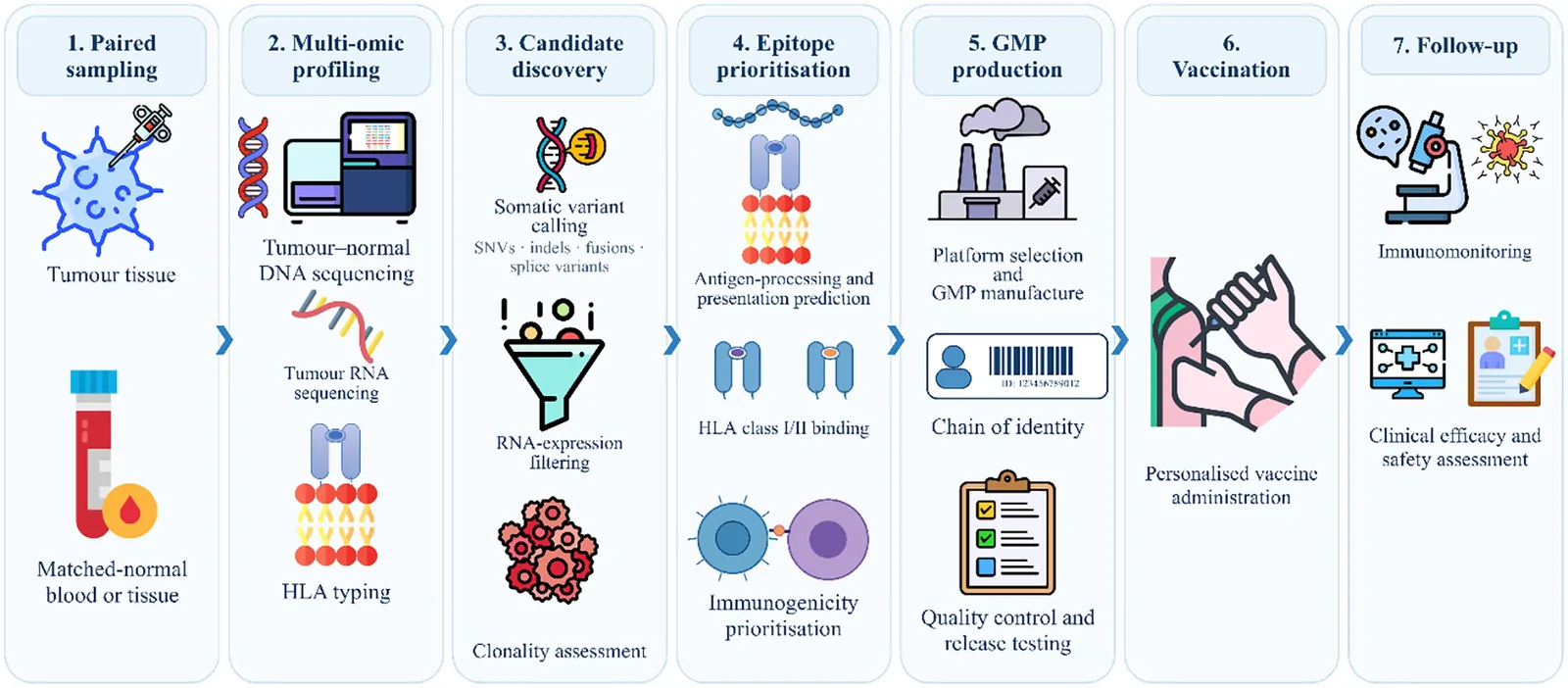
End-to-end workflow for personalised neoantigen vaccine development, from paired tumour and matched-normal sampling through multi-omic profiling, candidate discovery, epitope prioritisation, GMP production, vaccination, and post-vaccination immunological and clinical monitoring.

The development of a personalised neoantigen vaccine is a bespoke, time-sensitive and platform-dependent process integrating tumour genomics, computational immunology and GMP-compliant pharmaceutical production (40). The workflow begins with paired tumour and matched-normal blood or tissue sampling, followed by tumour and matched-normal DNA sequencing, tumour RNA sequencing and high-resolution HLA typing. Bioinformatic pipelines identify tumour-specific alterations, including nonsynonymous single-nucleotide variants, insertions and deletions, frameshifts, gene fusions and aberrant splice events. Candidate alterations are subsequently filtered according to tumour RNA expression, variant allele frequency, clonality and technical confidence. The resulting mutant peptides are evaluated for antigen processing and presentation, binding to the patient’s HLA class I and class II molecules, and their predicted capacity to elicit a T-cell response. The number of prioritised neoepitopes varies according to the vaccine platform, tumour type, trial protocol and manufacturing constraints; the selection of approximately 10–20 candidates represents an example used in some peptide- and RNA-based clinical studies rather than a universal standard. Selected candidates are manufactured as synthetic peptides, RNA or other vaccine formulations under GMP conditions, combined with an appropriate adjuvant or delivery system, and subjected to identity, purity, sterility, potency and other platform-specific release tests. Stringent chain-of-identity procedures must be maintained from sample collection through manufacture and administration. Vaccination schedules, including prime–boost regimens, are protocol dependent and should be accompanied by immunomonitoring and clinical safety and efficacy assessments. Reported manufacturing turnaround times of approximately 8–12 weeks apply to particular clinical platforms and workflows (41); actual timelines vary according to sequencing capacity, computational automation, manufacturing technology, regulatory requirements and institutional infrastructure. Scalability and reproducibility therefore remain constrained by complex variant interpretation, patient-specific manufacturing, rigorous quality-control requirements and the need to coordinate multiple clinical and production stages (**Figure 1**).

## Bioinformatics and AI-assisted neoantigen prediction

The primary reports for ImmuneMirror, NeoaPred, HLAIImaster and CNNeoPP do not provide prespecified, prospective, allele-stratified validation in Southeast Asian clinical cohorts (**Table 2**). ImmuneMirror reported an internal test AUC of 0.87 using neopeptides curated from 19 studies and applied the pipeline to 805 gastrointestinal tumours, but this is not regional clinical validation (6). NeoaPred is an HLA class I structural/immunogenicity model evaluated on retrospective datasets; its paper does not establish clinical utility in Southeast Asian alleles (37). HLAIImaster used 223,931 HLA class II eluted-ligand pairs across 72 alleles and a separate immunogenicity dataset, but no Southeast Asian prospective validation was reported (38). CNNeoPP is also HLA class I focused: it trained on 1,498 curated peptides, used an independent 153-peptide set and five public TESLA samples, and performed experimental work in three patients; these results support proof of concept, not broad clinical feasibility (39). Future regional validation should report results by allele and HLA locus, compare class I and class II tasks separately, and publish both positive and negative immunopeptidomic and T-cell assay results.

**Table 2:** Neoantigen-prediction tools classified by biological task, evidence base and validation status.

Tool/version	Primary task	HLA class	Training/evaluation evidence	Availability	Key limitation for this Review
*NetMHCpan 4.1* (34)	Peptide binding/eluted-ligand presentation score	I	Integrates binding-affinity and mass-spectrometry eluted-ligand data; independent epitope/MS benchmarks.	Web server; academic binaries	Not a T-cell immunogenicity or clinical-outcome model; allele coverage and assay composition must be reported.
*NetMHCIIpan 4.0* (34)	Peptide binding/presentation	II (DR, DQ, DP)	Binding-affinity and eluted-ligand data with motif deconvolution; version-specific datasets.	Web server; academic use	Class II peptide length, multi-allelic deconvolution and sparse allele data complicate comparison; not prospective clinical utility.
*MHCflurry 2.0* (35)	Binding, processing and integrated presentation	I	Binding and mass-spectrometry ligands; processing model separates allele-independent processing from binding.	Open source	Presentation probability is not equivalent to immunogenicity; performance metrics are not directly comparable across datasets.
*pVACtools 7.1.1* (36)	Workflow orchestration and therapy design	I/II via integrated predictors	No single training dataset: integrates variant/expression inputs and multiple predictors/filters.	Open source; document exact version, databases and predictor releases	A workflow, not a single AI predictor; outputs inherit limitations of variant calls, HLA typing and component models.
*ImmuneMirror 1.0* (6)	Multi-omic candidate prioritisation	Primarily I; pipeline reports broader features	Balanced random forest trained/tested on immunogenic neopeptides curated from 19 studies; internal test AUC 0.87; applied to 805 GI tumours.	Open-source pipeline and web server	Retrospective/internal performance; no prespecified Southeast Asian allele-stratified prospective validation.
*NeoaPred (*2024*)* (37)	Structural peptide–HLA and immunogenicity scoring	I	Retrospective structure and immunogenicity datasets with independent tests reported by the authors.	Open-source code	No class II model and no demonstrated prospective utility in common Southeast Asian alleles; do not generalise aggregate metrics.
*HLAIImaster (*2024*)* (38)	Eluted-ligand presentation and immunogenicity	II	223,931 eluted-ligand pairs across 72 class II alleles; immunogenicity data included 4,384 positive and 3,469 negative candidates plus decoys; cross-validation/held-out analyses.	Open source/repository	No prespecified prospective Southeast Asian validation; computational metrics and clinical benefit are distinct.
*CNNeoPP (*2026*)* (39)	End-to-end HLA-I prioritisation using LLM-derived embeddings and multimodal features	I	1,498 curated training peptides; 153 independent candidates; five TESLA samples (532 peptides, 34 immunogenic; 8 recovered in top 50); experimental work in three patients.	Research code/database as reported	Proof of concept, not broad clinical feasibility; small experimental cohort, HLA-I focus and no Southeast Asian allele-stratified prospective validation.

AUC, area under the receiver operating characteristic curve; GI, gastrointestinal; HLA, human leukocyte antigen; LLM, large language model; MS, mass spectrometry. Metrics from different training/test sets are not directly comparable. No listed tool has established prospective clinical utility or prespecified allele-stratified validation across representative Southeast Asian HLA class I and II repertoires. For reproducibility, report tool version or Git commit, reference database release, HLA-typing method, component predictors and access date.

### Vaccine platforms and delivery technologies

The choice of vaccine platform strongly influences immunogenicity, manufacturing requirements, turnaround time, scalability and cost. Synthetic long-peptide vaccines have been extensively investigated and offer chemical stability and the capacity to incorporate multiple neoepitopes; however, they commonly require potent adjuvants and appropriate delivery systems to induce robust cellular immune responses. mRNA vaccines have gained prominence because of their flexible design, relatively rapid synthesis and innate immune-stimulating properties. Experience gained from mRNA-based SARS-CoV-2 vaccines has advanced RNA design, lipid-nanoparticle formulation, analytical testing, GMP manufacturing and cold-chain infrastructure, thereby providing a valuable foundation for oncology applications (42). Nevertheless, these pathways are not directly transferable to personalised cancer vaccines. Individualised products are typically manufactured as small, patient-specific batches and require stringent chain-of-identity controls, rapid design-to-release workflows, product-specific quality-control and release testing, specialised dose formulation, and distinct regulatory oversight. Emerging nanocarrier systems may improve nucleic-acid stability, cellular delivery and immunogenicity, although evidence that these technologies can reliably eliminate cold-chain requirements remains limited (43). Across all platforms, a central challenge is balancing the degree of personalisation against manufacturing complexity, turnaround time and cost—a trade-off that is particularly important in resource-constrained health systems.

### Manufacturing, scalability, and translational readiness

The personalised nature of neoantigen vaccines upends conventional batch manufacturing, demanding stringent chain-of-identity controls, rapid release testing, and regulatory frameworks capable of accommodating patient-specific therapeutics. Turnaround time, cost, cold-chain dependencies, and the absence of harmonised approval pathways—particularly within the ASEAN region—collectively redefine the challenge as one of implementation science (41, 44).

### Biological determinants of vaccine response and failure

A predicted neoepitope cannot mediate tumour control if the target is absent from clinically relevant tumour clones, is not presented, or encounters dysfunctional effector cells. Tumour evolution can delete the presenting HLA allele through HLA loss of heterozygosity; in non-small-cell lung cancer, allele-specific HLA loss was reported in 40% of tumours and was associated with immune selection (45). Loss or downregulation of B2M, TAP1/TAP2, tapasin, immunoproteasome components or the MHC class I transactivator NLRC5 can impair peptide loading or surface presentation, whereas JAK1/JAK2 pathway defects can blunt interferon-γ signalling; B2M and JAK1/JAK2 alterations have been observed in acquired resistance to PD-1 blockade (46). Even when presentation is intact, chronic antigen exposure and co-inhibitory pathways including PD-1, LAG-3 and TIM-3 can constrain vaccine-expanded T cells. Regulatory T cells, myeloid-derived suppressor cells, suppressive cytokines, hypoxia and poor T-cell trafficking provide additional barriers. Clinical programmes should therefore measure target clonality and expression, HLA integrity and antigen-presentation machinery, tumour immune contexture, and longitudinal neoantigen-specific T-cell function rather than relying on peripheral immunogenicity alone.

## Clinical evidence and emerging therapeutic applications

### Early clinical development

Clinical evidence has progressed from first-in-human feasibility studies to randomised testing, although it remains heterogeneous by platform, disease setting and concomitant therapy (**Table 3**). Early personalised peptide and RNA studies in melanoma and glioblastoma established manufacturability and induction of neoantigen-specific CD4+ and CD8+ T-cell responses (47–50). The open-label, randomised phase 2b KEYNOTE-942 study subsequently assigned 157 patients with completely resected stage IIIB–IV cutaneous melanoma to V940 (mRNA-4157) plus pembrolizumab (n=107) or pembrolizumab alone (n=50) (51). At the primary analysis, recurrence-free survival favoured the combination (hazard ratio 0.561, 95% confidence interval 0.309–1.017; two-sided P = 0.053), with 18-month recurrence-free survival of 79% versus 62%. Grade 3 or higher treatment-related adverse events occurred in 25% and 18%, respectively. The combination design does not isolate the vaccine’s independent effect, and confirmation in larger randomised studies and additional tumour types remains necessary.

**Table 3:** Selected prospective clinical studies of personalised neoantigen vaccines, ordered by evidence maturity.

Tumour/setting	Product and concomitant therapy	Design (n)	Main published finding	Critical limitation
Resected high-risk melanoma (47)	Personalised synthetic long-peptide vaccine plus poly-ICLC	Phase I (6 vaccinated)	Feasible and immunogenic; induced polyfunctional neoantigen-specific CD4+ and CD8+ responses.	Very small, nonrandomised study; not designed for efficacy.
Stage III/IV melanoma (48)	Personalised RNA mutanome vaccine	First-in-human study (13)	Poly-specific T-cell responses were induced in all patients; tumour infiltration was shown in selected cases.	Small, heterogeneous, nonrandomised cohort; subsequent therapies confound clinical outcomes.
Newly diagnosed glioblastoma (49)	Personalised long-peptide neoantigen vaccine plus poly-ICLC with standard therapy	Phase Ib (8 vaccinated)	Circulating and intratumoural neoantigen-specific T-cell responses were observed, particularly without dexamethasone exposure.	Very small cohort; steroid and standard-treatment confounding; no randomised efficacy test.
Resected pancreatic ductal adenocarcinoma (52, 53)	Autogene cevumeran (individualised uridine-mRNA–lipoplex vaccine) with atezolizumab and modified FOLFIRINOX	Phase I; 19 safety-evaluable, 16 vaccinated/biomarker-evaluable	8 of 16 developed high-magnitude vaccine-induced responses; at 3.2-year follow-up, responder median RFS was not reached versus 13.4 months in nonresponders.	Nonrandomised multimodal regimen; responder analysis is associative and vulnerable to selection/immortal-time biases despite landmark analyses.
Completely resected stage IIIB–IV cutaneous melanoma (51)	V940 (mRNA-4157) plus pembrolizumab vs pembrolizumab	Open-label randomised phase 2b (157; 107 vs 50)	RFS HR 0.561 (95% CI 0.309–1.017; P = 0.053); 18-month RFS 79% vs 62%.	Open label; combination design cannot isolate vaccine effect; modest sample; confirmatory evidence required.
Advanced hepatocellular carcinoma after multikinase inhibitor (54)	GNOS-PV02 DNA vaccine encoding up to 40 neoantigens plus plasmid IL-12 and pembrolizumab	Single-arm open-label phase I/II (36)	ORR 30.6% (11/36); neoantigen-specific T-cell responses increased in 19/22 evaluable patients.	Single arm with pembrolizumab; historical comparisons cannot establish vaccine contribution.
High-risk resected clear-cell renal-cell carcinoma (55)	Personalised long-peptide vaccine with or without local low-dose ipilimumab	Phase I (9)	All patients mounted vaccine-specific T-cell responses; no recurrence at median 40.2 months after surgery.	Single-centre, very small, nonrandomised cohort; clinical outcome is hypothesis-generating.

CI, confidence interval; HR, hazard ratio; IL-12, interleukin-12; ORR, objective response rate; RFS, recurrence-free survival. Immunogenicity and within-cohort responder associations do not establish clinical efficacy. Table entries are based on primary reports, not secondary reviews.

### Melanoma

Melanoma provided the first clinical demonstrations that personalised peptide and RNA vaccines can generate broad *de novo* and boosted neoantigen-specific T-cell responses (47, 48). Evidence has since advanced beyond single-arm feasibility. In KEYNOTE-942, V940 plus pembrolizumab was compared with pembrolizumab alone after complete resection of high-risk cutaneous melanoma (51). The recurrence-free-survival signal supports further development, but the open-label combination design, sample size and P value at the primary analysis warrant calibrated interpretation. The trial establishes randomised comparative evidence for the regimen; it does not prove the independent contribution of V940 or generalisability to other tumour types or lower-resource health systems.

### Lung cancer

Non-small cell lung cancer (NSCLC) presents an attractive target owing to often high mutational burdens and an established role for checkpoint inhibitors. Kyriakidis et al., 2025 (56) reviewed next-generation combination regimens and concluded that neoantigen vaccines can amplify the depth and durability of checkpoint inhibitor responses. Yaremenko et al., 2025 (57) highlighted the parallel development of mRNA therapeutic platforms for lung cancer. However, patient attrition during the manufacturing window is a significant concern in metastatic disease, and the clonal heterogeneity of lung tumours may lead to immune escape.

### Glioblastoma

In newly diagnosed glioblastoma, a phase Ib personalised long-peptide vaccine study demonstrated circulating and intratumoural neoantigen-specific T-cell responses in vaccinated patients, with reduced responses in the setting of dexamethasone exposure (49). A separate actively personalised vaccination study integrated premanufactured tumour-associated antigens and patient-specific neoepitopes (50). These studies establish feasibility and immune trafficking but are too small and confounded by concurrent standard therapy to determine survival benefit. Steroid exposure, lymphopenia, spatial heterogeneity, antigen-presentation loss and the immunosuppressive central-nervous-system microenvironment remain major barriers.

### Pancreatic and hepatobiliary cancers

In resected pancreatic ductal adenocarcinoma, autogene cevumeran induced high-magnitude T-cell responses in 8 of 16 vaccinated patients in a phase I multimodal study (52). At 3.2 years, vaccine responders had durable CD8+ T-cell clones and longer recurrence-free survival than nonresponders, but this within-cohort association cannot establish causality (53). In advanced hepatocellular carcinoma, a single-arm phase I/II study combined the personalised DNA vaccine GNOS-PV02, plasmid-encoded interleukin-12 and pembrolizumab in 36 patients; the objective response rate was 30.6%, and neoantigen-specific T-cell responses increased in 19 of 22 evaluable patients (54). Because the HCC study lacked a concurrent control and used combination therapy, the vaccine’s incremental clinical effect remains uncertain. Viral aetiology, cirrhosis, antigen-presentation capacity and hepatic immune tolerance should be prespecified in future regional studies.

### Combination strategies

It is increasingly apparent that personalised neoantigen vaccines will realise their full potential not as standalone agents, but as integral components of rationally designed combination regimens. The synergy between neoantigen vaccination and checkpoint blockade is mechanistically compelling: vaccines expand the pool of tumour-reactive T cells, while anti-PD-1 or anti-CTLA-4 antibodies prevent their functional exhaustion (56, 58). Chemotherapy and radiotherapy can further complement vaccine activity by inducing immunogenic tumour cell death and depleting suppressive immune populations. Mansinho et al., 2026 (59) discussed the convergence of oncolytic virus therapy and neoantigen vaccination, noting that virally induced tumour lysis provides an *in situ* source of neoantigens. Despite this rationale, clinical data on optimal sequencing and biomarker-guided selection are lacking, and the additive toxicities require meticulous evaluation.

## Infrastructure, economic, and translational challenges in Southeast Asia

### Genomic and molecular diagnostic infrastructure

**Figure 2:**
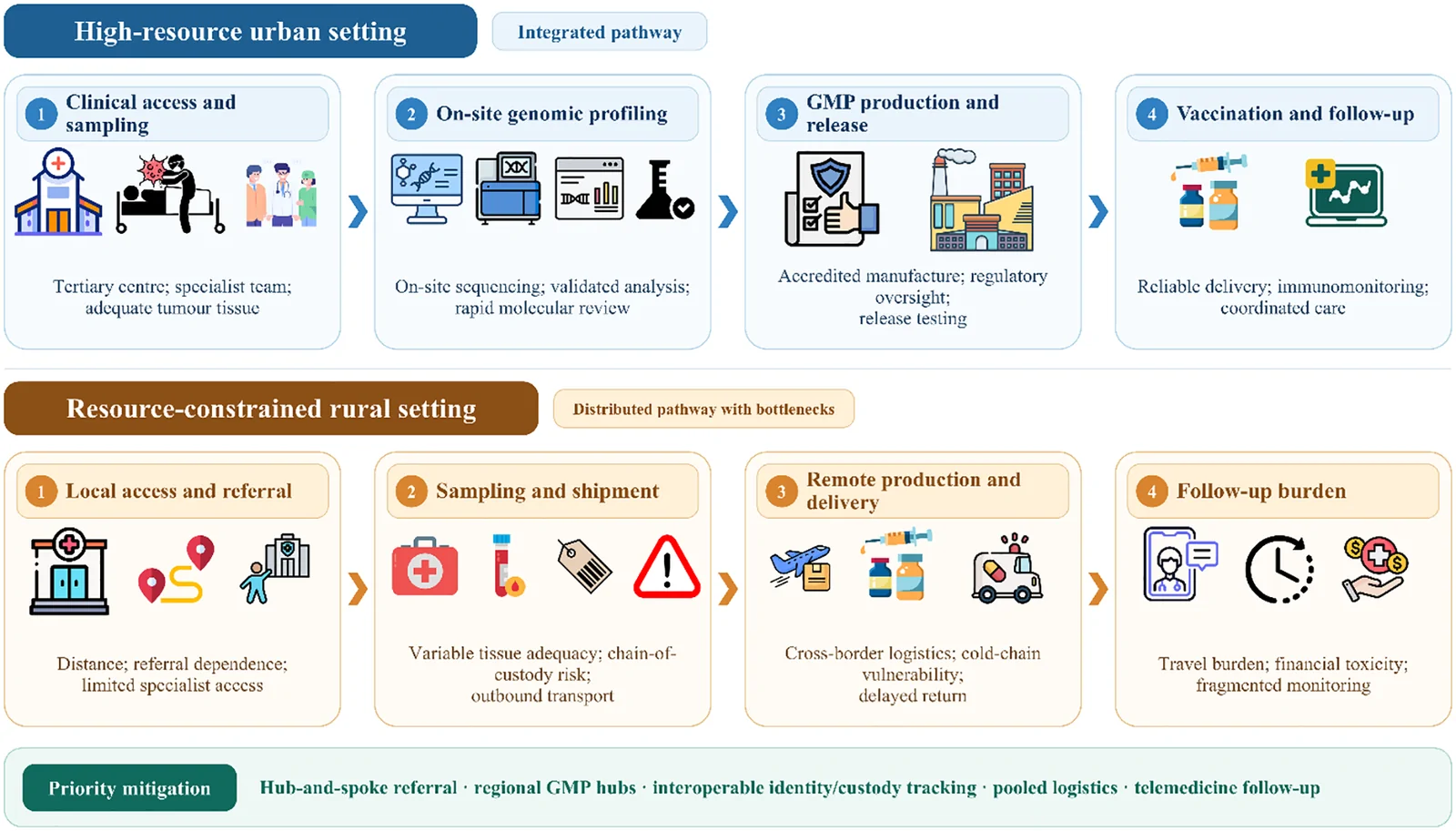
Healthcare inequity and translational bottlenecks for personalised cancer vaccines in Southeast Asia.

The foundational step of comprehensive tumour genomic profiling remains inaccessible for most cancer patients in Southeast Asia. Advanced molecular diagnostics such as NGS are concentrated in a handful of urban academic centres, while public hospitals often lack even reliable immunohistochemistry (7). Even in Thailand, an upper-middle-income country, NGS utilisation is confined largely to research settings (18). In Indonesia, national health insurance has improved coverage for basic oncology services but has not resolved the structural deficit in molecular pathology and genomic sequencing capacity (10). Quality assurance and standardisation remain unaddressed; external proficiency testing for NGS is inconsistently implemented, raising concerns about the reliability of neoantigen prediction if clinical-grade tumour-normal sequencing is not routinely achievable. The absence of accredited biobanking networks further hampers the collection of paired tumour and germline specimens, a prerequisite for refining region-specific immunogenomic algorithms (60) (**Figure 2**).

### Economic barriers and financial toxicity

The personalised nature of neoantigen vaccines introduces a cost structure fundamentally incompatible with the constrained health budgets of Southeast Asia. The cumulative expense has been estimated to range from tens to over a hundred thousand US dollars per patient in high-income settings (41). Feliciano et al., 2025 (61) specifically examined barriers to immune checkpoint inhibitor access in Southeast Asia and identified prohibitive drug costs, absence of companion diagnostics, and lack of health technology assessment capacity as primary obstacles—all of which would apply with even greater force to a patient-specific therapeutic. Many ASEAN countries have expanded universal health coverage schemes, yet these programmes commonly exclude high-cost biologics and personalised medicines, leaving patients to shoulder catastrophic out-of-pocket payments (10). The absence of region-specific cost-effectiveness analyses for personalised cancer vaccines, combined with the methodological challenges of health economic modelling for patient-specific interventions (62). means that payers lack the evidence base to make informed coverage decisions.

### Manufacturing and supply chain challenges

Personalised neoantigen vaccines challenge conventional pharmaceutical production because each product is designed and manufactured for an individual patient, requiring small-batch production, rapid turnaround and continuous chain-of-identity control. Although mRNA platforms offer flexible design and comparatively rapid synthesis, their storage requirements are formulation and product specific. Depending on lipid composition, stability data, intended shelf-life and manufacturing process, products may require refrigerated, frozen or ultra-low-temperature storage; therefore, a universal requirement of −20 °C or below should not be assumed (43, 44). In parts of Southeast Asia, reliable temperature-controlled transport, continuous monitoring, backup power and last-mile delivery may nevertheless present substantial challenges, particularly outside major urban and tertiary-care centres.

Similarly, the frequently cited interval of 8–12 weeks from biopsy to first administration reflects particular clinical platforms and manufacturing workflows rather than a universal production timeline. Turnaround depends on sample acquisition, sequencing capacity, bioinformatic analysis, regulatory review, manufacturing technology, quality-control testing and product release. Cross-border shipment of specimens or patient-specific products may introduce additional delays associated with transport permits, customs clearance and temperature-controlled logistics. Such delays are clinically important for patients with rapidly progressing malignancies.

Quality-control and release testing are also complicated by the manufacture of small, patient-specific batches. Conventional statistical sampling approaches used for large commercial batches may be unsuitable, necessitating validated rapid assays, risk-based release specifications and platform-specific testing for identity, purity, potency, sterility and product integrity. Regulatory requirements differ among Southeast Asian jurisdictions, and the applicability of existing frameworks for biologics, advanced therapies and investigational products to individualised neoantigen vaccines should be evaluated on a country-specific basis rather than characterised as uniformly absent. Dependence on imported sequencing reagents, synthesis materials, lipid components and specialised equipment also varies by country and should be supported by current national evidence rather than broad regional generalisations.

A practical implementation option is a regional hub-and-spoke model. Tertiary hospitals and cancer centres could serve as clinical spokes responsible for patient selection, paired tumour and matched-normal sampling, local sequencing where available, vaccine administration and immunomonitoring. Accredited regional GMP hubs could undertake computational prioritisation, platform-specific manufacture, quality-control testing and batch release through shared quality agreements and interoperable chain-of-identity systems. Where regulatory and data-governance requirements permit, secure transfer of validated genomic and manufacturing data rather than repeated cross-border shipment of biological specimens could reduce delays. The feasibility of this model would depend on technology transfer, workforce development, regulatory recognition, sustainable procurement and clearly defined responsibility for product release and pharmacovigilance.

### Bioinformatics and AI infrastructure gaps

The computational pipeline that transforms raw sequencing data into a prioritised list of vaccine-eligible neoepitopes is arguably the weakest link in the translational chain for much of the global south. Tan et al. (63) made a compelling case for embedding data scientists within tumour boards, but simultaneously documented the acute shortage of bioinformaticians and clinical data analysts in low-resource settings. High-performance computing clusters and interoperable bioinformatics platforms are concentrated in a few academic hubs; outside these enclaves, genomic data analysis is frequently performed on desktop computers using *ad hoc* pipelines lacking standardisation. The deployment of advanced AI-driven tools demands computational resources and technical expertise unavailable in most regional hospitals. Moreover, Southeast Asian populations are substantially underrepresented in the training data for current neoantigen prediction algorithms, raising concerns about algorithmic bias (5, 64). The absence of federated data-sharing frameworks across ASEAN nations further fragments the genomic data that do exist, preventing the aggregation of sufficiently large, population-representative training cohorts.

### Workforce and human resource constraints

The delivery of personalised neoantigen vaccines demands a multidisciplinary team extending well beyond the traditional oncologist-pathologist dyad. Southeast Asia faces an across-the-board shortage of these cadres. A broader pattern of allied health workforce gaps extends to molecular pathology and precision oncology (65). The pipeline of genomic scientists and computational biologists is constrained by limited postgraduate training programmes and emigration of skilled professionals to high-income countries. Huq et al., 2024 (66) identified fragmented curricula and insufficient hands-on exposure to genomic technologies as persistent barriers, findings that resonate across Southeast Asian nations with comparable levels of economic development. Even in Singapore, which possesses the region’s most advanced precision oncology ecosystem, workforce-capacity constraints contribute to diagnostic and treatment delays (19). The coordination burden of personalised vaccine programmes would strain already overstretched clinical services and expose the absence of dedicated precision oncology navigators. Without a concerted regional strategy to expand training and retain talent, the human resource deficit will constitute a rate-limiting factor that no technological innovation can circumvent.

### Regulatory, ethical, and governance challenges

Personalised neoantigen vaccines exist in a regulatory grey zone that challenges the existing frameworks of drug approval, pharmacovigilance, and quality assurance. Within the ASEAN region, regulatory harmonisation remains nascent; there is no regional consensus on the oversight of patient-specific biologics or mRNA-based therapeutics (9). National regulatory authorities vary widely in their technical capacity to evaluate advanced therapy medicinal products, creating a patchwork of divergent requirements that would complicate multi-country clinical trials and equitable access. Ethical governance gaps further compound regulatory uncertainty. The generation, storage, and cross-border sharing of whole-exome and transcriptome data raise unresolved questions about genomic privacy, informed consent for secondary research uses, and the return of incidental findings. The potential use of AI algorithms in clinical decision-making introduces additional concerns regarding algorithmic transparency, accountability, and the risk of encoding existing healthcare disparities into automated tools (64). Without proactive development of region-specific ethical guidelines and regulatory pathways, the introduction of personalised neoantigen vaccines risks proceeding in a governance vacuum that could undermine public trust and exacerbate inequities.

### Healthcare inequities and translational feasibility

The infrastructure and economic barriers enumerated above intersect with and amplify pre-existing axes of healthcare inequity. Alberto et al., 2023 (7) documented severe disparities in access to cancer diagnostics along economic, geographic, and educational gradients. Arevalo et al., 2023 (67) demonstrated that sex disparities in cancer outcomes are shaped by social determinants that would likely influence access to personalised immunotherapy. Segelov, 2023 (68) emphasised that equity must be placed at the centre of the cancer care continuum in the Asia-Pacific, yet the current trajectory of personalised medicine development—market-driven, urban-centric, and technologically intensive—risks creating a two-tier system. Barragan-Carrillo et al., 2025 (69) argued that direct transplantation of high-income-country precision oncology models into LMIC settings is neither feasible nor desirable. Lajmi et al., 2024 (70) identified the absence of system-wide implementation frameworks as a critical barrier to precision oncology adoption, a finding that resonates strongly in the ASEAN context, where national cancer control plans rarely incorporate genomic medicine. The mixed public–private financing landscape creates perverse incentives: private providers may offer NGS and targeted therapies to a wealthy minority, while public-sector investment in the infrastructure required for equitable, population-scale precision oncology remains politically unattractive. Near-term implementation of personalised neoantigen vaccines in Southeast Asia is therefore unlikely to follow the paradigm of comprehensive, fully individualised manufacture; rather, a phased approach that prioritises shared neoantigens, simplified mRNA constructs, and regional manufacturing hubs may offer a more realistic pathway.

## Policy perspectives and regional strategies

### Strengthening regional genomic and precision oncology infrastructure

Any aspiration to deliver personalised neoantigen vaccines must begin with concerted investment in foundational diagnostic and computational infrastructure. The extreme disparities in access to NGS and molecular pathology demand a deliberate strategy of decentralisation through tiered laboratory networks linking tertiary referral centres with provincial hospitals. The Thailand precision medicine scoping review demonstrated that even modest public investment can catalyse the establishment of institutional NGS capacity (18). Critically, these investments must encompass not only sequencers but also accredited biobanking facilities, standardised bioinformatics pipelines, and interoperable health information systems that enable longitudinal clinical-genomic data linkage.

### Regional collaborative networks and multicentre research

The near-total absence of neoantigen vaccine clinical trial activity in Southeast Asia is both a symptom of infrastructural weakness and a cause of ongoing exclusion from evidence generation. Regional collaborative networks modelled on existing initiatives such as the Asian Lymphoma Study Group provide proof-of-principle that cross-border oncology research is feasible (71). Extending this model to precision immunotherapy would require harmonised clinical trial protocols, shared immunomonitoring platforms, and a regional biobanking consortium that prospectively collects paired tumour-normal specimens from ethnically diverse populations. Multicentre validation studies of neoantigen prediction algorithms using Southeast Asian genomes are an urgent scientific priority; without them, the performance of AI tools in local populations will remain uncertain, and regulators will lack the evidence base to authorise clinical use.

A staged ASEAN hub-and-spoke model could align technical complexity with existing capacity. Local clinical spokes would identify eligible patients, obtain consent, collect tumour and matched-normal specimens, stabilise and ship material, and maintain chain-of-identity records. Accredited national nodes would perform pathology review, tumour–normal DNA sequencing, tumour RNA sequencing, HLA typing, validated bioinformatics, molecular tumour-board review and immunomonitoring. A limited number of regional GMP hubs could undertake construct synthesis, formulation, fill–finish, identity/purity/potency testing, sterility and endotoxin testing, lot release, and validated return shipping. Common technical dossiers, release specifications, audit trails, cross-border material-transfer agreements and data-governance templates would support regulatory reliance and joint scientific advice while preserving national legal authority. Decentralised manufacture should be introduced only when case volume, quality systems, workforce and regulator oversight can sustain equivalent GMP performance; bench-top or point-of-care systems remain investigational. Implementation studies should prospectively report biopsy-to-sequencing time, sequence-to-design time, manufacturing and release failures, chain-of-identity deviations, patient attrition, total biopsy-to-dose time, cost per completed course, and rural–urban access.

### Public–private partnerships and sustainable funding models

The prohibitive cost of personalised neoantigen vaccine manufacture necessitates innovative financing architectures. Public–private partnerships could de-risk the capital investments required for regional mRNA manufacturing facilities, with governments providing infrastructure and regulatory support and biotechnology partners contributing process development and quality-assurance expertise. International financing mechanisms, including multilateral development bank loans and global health funds, might be harnessed to subsidise the initial rollout of personalised vaccine programmes within public-sector oncology services. Aguiar et al. (62) outlined the methodological challenges of health economic modelling for personalised oncology, yet such analyses are precisely what regional health technology assessment agencies require to inform coverage decisions; their development should be prioritised through collaborative academic–government initiatives. A staged reimbursement model, in which vaccines are initially offered to patient subsets with the highest probability of benefit, could enable cost containment while evidence of real-world effectiveness is accumulated.

### Workforce development and capacity building

The expansion of precision oncology infrastructure must be matched by an equally ambitious investment in human capital. Tan et al., 2025 (63) argued that data scientists should be embedded within tumour boards in the global south, but the workforce pipeline remains critically underdeveloped. ASEAN-wide fellowship programmes in precision oncology, sponsored jointly by ministries of health and international professional societies, could create a cadre of specialists equipped to lead molecular tumour boards and oversee personalised vaccine programmes. Task-shifting strategies and retention incentives are equally critical to counter the brain drain that chronically depletes the region of its most skilled professionals. The educational reforms advocated by Huq et al. (66) for the SAARC region provide a blueprint for modernising curricula and strengthening continuing professional development in genomic medicine.

### AI, digital health, and emerging ecosystems

Artificial intelligence and digital health technologies represent both a transformative opportunity and a potential amplifier of inequity. A regional federated learning network, in which genomic data remain securely within each country while model parameters are shared, could address privacy concerns while enabling the development of population-calibrated prediction tools (64). Cloud-based genomic analytics platforms, if subsidised and made interoperable with national health information systems, could democratise access to advanced bioinformatics without requiring every hospital to maintain on-premises high-performance computing. Digital pathology and tele-oncology networks could further extend the reach of precision diagnostics to underserved areas, provided that digital literacy and internet connectivity are concurrently strengthened.

### Regulatory harmonisation and ethical governance

ASEAN member states should work toward a mutual recognition framework for advanced therapy medicinal products that establishes common standards for GMP compliance, quality-control testing, and post-market surveillance of personalised vaccines. This framework could be informed by the regulatory flexibilities adopted during the COVID-19 pandemic, which demonstrated that accelerated, conditional approval pathways can be implemented without compromising safety when accompanied by robust pharmacovigilance. Ethical governance must be addressed with equal urgency: region-specific guidelines on informed consent, data sovereignty, and the return of incidental findings are essential, as is the development of institutional review board expertise in reviewing protocols that involve AI-assisted clinical decision-making.

### Equity, accessibility, and integration into public healthcare systems

The most meticulously designed policy framework will fail if it does not deliberately centre equity. Feliciano et al. (61) documented that even immune checkpoint inhibitors remain inaccessible to most patients in Southeast Asia. Proactive measures are essential: tiered pricing agreements with manufacturers, compulsory licensing provisions for enabling technologies, and the inclusion of personalised vaccines in national essential medicines lists once efficacy is established. Integration into universal health coverage schemes must be accompanied by robust health technology assessment and budgetary impact analyses that inform prioritisation decisions in a transparent, accountable manner (62). Rural outreach can be enhanced through mobile diagnostic units, decentralised biopsy services, and telemedicine follow-up that reduces the travel burden on patients. Context-appropriate, phased implementation—rather than wholesale adoption of high-income-country models—is the most viable path to equitable access (69).

## Future directions

Looking ahead, several technological and translational developments could substantially improve the feasibility of personalised neoantigen vaccines in resource-limited settings. Decentralised, bench-top mRNA synthesis platforms could reduce dependency on centralised contract manufacturing organisations and shorten turnaround times (42, 44). The identification of shared neoantigens arising from recurrent driver mutations—such as those in *KRAS*, *TP53*, or *EGFR*—raises the possibility of semi-off-the-shelf vaccines that retain a degree of personalisation without the full complexity of bespoke manufacture (72). Bioinspired cell-membrane-coated nanovaccines could simplify formulation and enhance immunogenicity (73). Realistically, these innovations are five to ten years from clinical maturity, and their translation into Southeast Asian healthcare systems will require the foundational investments in genomics, workforce, and regulatory capacity described above. The region must therefore pursue a dual-track strategy: addressing immediate infrastructure deficits while positioning itself to absorb and adapt emerging technologies as they become validated.

### Convergent enabling technologies

Emerging advances in cellular immunology and tumour biology may inform future personalised neoantigen-vaccine strategies. Memory T-cell subsets possess self-renewal, persistence and recall-response properties that are important for durable adoptive immunotherapy and may also guide the evaluation of long-lived vaccine-induced T-cell responses (74). Meanwhile, liquid–liquid phase separation regulates oncogenic transcription, DNA repair, signal transduction and treatment resistance, suggesting that tumour-cell state may influence antigen expression and immune responsiveness (75).

Materials engineering provides additional principles for vaccine delivery and rational combination therapy. Molecular tailoring of doxorubicin prodrugs can improve nanoassembly stability and tumour-responsive drug activation (76), while arsenic trioxide-based nanoparticles can enhance tumour-cell killing through pyroptosis and stimulate antitumour immune responses (77). In another biomedical context, an aggregation-induced-emission-luminogen system developed for mpox demonstrated stimulus-responsive disease monitoring and localised treatment, illustrating the broader potential of intelligent theranostic materials (78). Although these studies do not directly validate personalised neoantigen vaccines, they support future investigation of stable nanocarriers, immunogenic-cell-death combinations, durable T-cell responses and treatment-responsive monitoring systems.

## Conclusion

The advent of personalised neoantigen vaccines marks a defining moment in precision oncology. By targeting the unique repertoire of somatic mutations, these bespoke immunotherapies offer a mechanism for tumour eradication that is both exquisitely specific and adaptable to individual patients. Convergent advances in sequencing, AI-driven neoantigen prediction (79), and mRNA delivery (42) have transformed neoepitope identification into a clinically feasible workflow. Early clinical experiences have confirmed safety, immunogenicity, and, in select patients, durable disease control.

Yet the distance between technological capability and equitable clinical reality is vast, particularly within the heterogeneous health systems of Southeast Asia. The region’s cancer burden, exceeding 1.1 million new cases annually, is rising against a backdrop of inadequate genomic diagnostic infrastructure, severe workforce shortages, and health budgets strained by competing priorities (1, 7). The bespoke manufacturing model, with its attendant costs and cold-chain dependencies, is fundamentally misaligned with the resource constraints of public-sector oncology services. Furthermore, the regulatory and ethical governance frameworks required for patient-specific advanced therapies are embryonic. Without proactive remediation, these deficits will relegate personalised neoantigen vaccines to a niche intervention available only through private centres of excellence, thereby amplifying the profound cancer care inequities already documented in the region.

A deliberate, multi-pronged strategy is therefore imperative. Regional precision oncology networks, anchored by centres of excellence and linked through harmonised protocols, can accelerate locally relevant clinical research while building diagnostic and immunomonitoring capacity. Investments in cloud-based bioinformatics, federated learning platforms, and population-representative immunogenomic databases are critical to ensuring that neoantigen prediction algorithms perform equitably across Southeast Asia’s ethnically diverse populations. Public–private partnerships and innovative financing mechanisms can reduce the capital barriers to regional mRNA manufacturing, while parallel workforce development—through ASEAN-wide fellowship programmes and curriculum modernisation—must address the brain drain that chronically weakens the region’s scientific cadres.

The question confronting Southeast Asia is not whether personalised neoantigen vaccines will eventually become part of the oncology armamentarium, but whether their introduction will narrow or widen the chasm of cancer inequity. The answer will depend on the political determination to construct the translational ecosystems, regulatory compacts, and financing mechanisms that can deliver these innovations to all patients, irrespective of geography or income. The convergence of scientific possibility and unmet clinical need in this dynamic region presents a historic opportunity; seizing it demands that the architects of precision medicine commit to a shared vision in which genomic innovation serves as a bridge to health equity, not a barrier.
